# Comparison of five DNA extraction methods in three medicinal plants: *Peganum harmala* L., *Tamarix ramosissima* Ledeb., and *Potentilla reptans* L. 

**DOI:** 10.22099/mbrc.2023.45131.1798

**Published:** 2023

**Authors:** Zahra Salehi, Atefe Amirahmadi, Arezou Rezaei, Parisa Farrokh, Javad Ghasemian

**Affiliations:** 1 School of Biology, Damghan University, Damghan, Semnan, Iran; 2 Institute of Biological Sciences, Damghan University, Damghan, Semnan, Iran; 3 School of mathematics and computer sciences, Damghan University, Damghan, Semnan, Iran

**Keywords:** Peganum harmala L., Tamarix ramosissima Ledeb., Potentilla reptans L., DNA extraction, CTAB, Murray and Thompson method

## Abstract

Extracting high-yield, high-quality DNA from plant samples is challenging due to the presence of the cell wall, pigments, and some secondary metabolites. The main CTAB method, two of its modified protocols (beta-mercaptoethanol or ammonium acetate were eliminated), the modified Murray and Thompson method, and the Gene All kit were statistically compared based on the quantity and quality of the total DNA (tDNA) extracted from fresh and dried leaves of three medicinal herbs *P. harmala*, *T. ramosissima*, and *P. reptans*. The suitability of the tDNAs for molecular studies was evaluated by polymerase chain reaction (PCR) of the fragments of the internal transcribed spacer (ITS) in nuclear DNA and the *trn*L-F region in chloroplast DNA. Some significant differences were found between the tDNAs extracted by five extraction methods. With the exception of *P. harmala*, where the PCR of both the ITS fragments and the *trn*L-F region worked successfully in all DNA samples, but only the ITS fragments, not the chloroplast *trn*L-F region, were amplified in the DNA samples of *T. ramosissima* and *P. reptans*. The chloroplast *trn*L-F region was amplified only in DNA samples extracted from fresh and dried leaves of the three studied herbs using the commercial kit. Gene All kit, the main CTAB method, and its modified protocols were the less time-consuming protocols that yielded DNA suitable for downstream PCR vis-a-vis the modified Murray and Thompson method.

## INTRODUCTION

Medicinal herbs and their products are of health and economic value. Extraction of high-quality DNA is required to study the genetic, morphological, and ecological characteristics of medicinal herbs [1]. The extraction of high-quality and high-yield DNA from plant samples is laborious due to the hard polysaccharide cell wall and secondary metabolites such as polysaccharides, polyphenols, alkaloids, and proteins [2-7], which can precipitate with DNA during extraction and inhibit DNA digestion and PCR [8]. Moreover, DNA extraction from medicinal herbs is often problematic in molecular studies as they are rich in polysaccharides or secondary metabolites such as polyphenols [9,10]. 

In the current study, three medicinally important herbs, including *Peganum harmala* L. (Zygophyllaceae), *Tamarix ramosissima* Ledeb. (Tamaricaceae), and *Potentilla reptans* L. (Rosaceae) were investigated. *P. harmala*, known as espand in Persian, is a perennial herbaceous plant native to the arid regions of North Africa, the Mediterranean, the Middle East, Pakistan, and India [11]. The herb is common in the provinces of Azerbaijan, Fars, Gorgan, Isfahan, Kerman, Khorasan, Khuzestan, Semnan, and Tehran in Iran [12]. Since ancient times, *P. harmala* has been traditionally and commonly used for medicinal and psychoactive purposes [11]. In Iran, *P. harmala* is traditionally used as an antiseptic by burning its seeds [13]. The herb is traditionally used in various countries to treat asthma, colic, fever, jaundice, backache, lice, and syphilis [14]. *P. harmala* has many other pharmacological properties, including analgesic, anticonvulsant, antihistamine, anti-inflammatory, antioxidant, antitumor [15, 16], antibacterial, antifungal, and antiviral activities [17]. *T*. *ramosissima*, called gaz in Persian, is a halophytic herb [18] that prefers alluvial soils but also grows well on saline and alkaline soils [19] and is widespread in the north, northwest, west, central, northeast, and east [20]. Because of its unparalleled flavor, *T. ramosissima* is used as a skewer [21]. The *Tamarix* species are useful in leucoderma, spleen disorders, eye diseases [19], and wound healing [22]. *T. ramosissima* has been shown to possess antibacterial, antioxidant, astringent, appetizing, and invigorating properties [22, 23]. *P. reptans* is a perennial plant from Caspian and Iranian-Turanian regions and is common in Afghanistan, Europe, Iran, Iraq, the former Soviet Union, Turkey, and North Africa. This herb is common in Azerbaijan, Boyer-Ahmad, Gorgan, Gilan, Kohgiluyeh, Lorestan, Semnan, and Tehran provinces of Iran [12, 24]. The genus *Potentilla* has been known for its therapeutic properties since ancient times. Extracts from the aerial or underground parts of *P. reptans* are used in traditional medicine against bacterial [25], fungal, and viral infections, cancer, diabetes, diarrhea, inflammation, and wounds [26]. 

Against this background, the present study aims to compare the quality of the extracted total DNA (tDNA) from fresh and herbarium samples of *P. harmala*, *T. ramosissima*, and *P. reptans* using five extraction methods: the main CTAB method [27], two modified CTAB methods, the modified Murray and Thompson method [28, 29], and the Gene All Kit method. To compare the quality of the extracted tDNAs for molecular studies, the internal transcribed spacer (ITS) region of 18S-26S nuclear ribosomal DNA (nrDNA) [30] and the *trn*L intron and *trn*L-F spacer in the chloroplast genome [31] were amplified by the polymerase chain reaction (PCR). Fragments of nrDNA ITS are the most important tool for phylogenetic studies in various taxonomy categories. ITS 4 and ITS 5m primers amplify the nrDNA ITS fragments. In phylogenetic studies of plants, the *trn*L-F region is one of the most common chloroplast markers. *trn*L(c) and *trn*L(f) primers are used to amplify specific sequences of tRNA genes in the chloroplast [32, 33].

## MATERIALS AND METHODS

**Herbs: **Fresh and dried leaves of *P. harmala*, *T. ramosissima*, and *P. reptans* were studied. *P. harmala* was collected on July 17, 2018, a Tuesday, on our way from Shahrood to Azadshar (Semnan province, Iran). On the same day, *T. ramossissima*, known commonly as salt cedar, was collected on our way from Damghan to Sari above Cheshmeh Ali (Semnan province). Finally, *P. reptans*, commonly known as creeping baby's breath, was collected on August 3, 2018, a Friday, from field margins in Sari (Mazandaran Province, Iran). Dr. Atefe Amirahmadi (Ph.D. in Plant Biosystematics) identified the plants. The specimens of *P. harmala* (Amirahmadi: 2048.), *T. ramosissima* (Amirahmadi: 2539.), and *P. reptans* (Amirahmadi: 2666.) were kept in the herbarium of Damghan University. The young leaves which were free of wounds and pests were isolated. Once they had been washed with distilled water, some of the leaves were frozen to -80°C, and the rest were dried at room temperature.

**DNA extraction:** Using five methods, the total DNA was extracted from dried and fresh (stored in the freezer) leaves. The three main extraction methods included the CTAB method [27], the modified Murray and Thompson method [28, 29] and the kit method. In addition, modifications were made to the original CTAB method to make DNA extraction more cost-effective. DNA extraction from all samples was performed in triplicate.

**Main and modified CTAB methods:** In the main CTAB method [27], 0.25 g of fresh plant leaves or 0.125 g of herbarium leaves were crushed with 3000 μL of a preheated CTAB buffer (60°C) in a preheated mortar, and 6 μL of beta-mercaptoethanol (BME; 0.2% extraction buffer) was added to the homogenate. The CTAB buffer contained 2% CTAB powder, 20 mM EDTA, 1.4 M sodium chloride, and 100 mM Tris-HCl (pH=7.5). Subsequently, 400 μL of homogenate were incubated in 1.5 ml microtubes for 30 minutes in a dry bath at 60°C. The homogenate was gently mixed every 5 minutes by turning the microtubes upside down. After 30 minutes, two volumes of chloroform-isoamyl alcohol (24:1) were added to the microtubes and mixed 20 times by slow inverting. The microtubes were centrifuged at 13000 rpm at 4°C for 15 minutes. The step was repeated when the supernatant was not clear. The upper aqueous phase containing tDNA was carefully transferred to new tubes, and two volumes of cold isopropanol were added to each microtube. The microtubes were centrifuged at 10,000 rpm at 4°C for 10 minutes after being kept at -20°C for 20 minutes. The supernatant was discarded, and the pellet was washed with 250 μL of a wash buffer (76% ethanol and 10 mM ammonium acetate). The microtubes were centrifuged at 10,000 rpm for 2 minutes. The wash buffer was discarded, and the pellet was dried on a paper towel at room temperature. The pellet was dissolved in 30 μL of deionized water. 

The first modified CTAB method differed from the main CTAB method in that no BME was used. In the second modified CTAB, only 76% ethanol without ammonium acetate was used to wash the DNA pellets. 

**The modified Murray and Thompson method: **In this study, the modified method of Murray and Thompson published by Riahi et al. in 2010 [29] was used. The components of the extraction buffer were 2% CTAB powder, 100 mM Tris-HCl (pH = 7.5), 1.4 M NaCl, and 50 mM EDTA (pH = 8). Fresh (0.25 g) leaves or 0.125 g of herbarium leaves were crushed in a mortar with 1875 μL of a CTAB buffer and 7.5 μL of BME was added to the homogenate. Then, 750 μL of the homogenate was transferred to new microtubes and incubated at 60°C in a dry bath for 1 hour, with careful mixing by inverting the microtubes every 15 minutes. In this step, 700 μL of chloroform-isoamyl alcohol (24:1) was added to all microtubes, and the contents were mixed by gently inverting the microtubes several times. The microtubes were centrifuged at 10,000 rpm, 4°C for 15 minutes. The upper aqueous phase containing the DNA was carefully transferred to new microtubes, and 0.33 volume of cold isopropanol was added to each microtube. The microtubes were kept at -20°C for 1 hour before centrifugation at 10,000 rpm and at 4°C for 15 minutes. The supernatant was discarded, and 100 μL of TE buffer (1 mM EDTA and 10 mM Tris), 0.1 volume of 2.5 M sodium oxaloacetate, and 2 volumes of 95% cold ethanol were added to the pellets. Samples were kept in the freezer at -20°C for 30 minutes. The microtubes were centrifuged at 10,000 rpm for 5 minutes. The supernatant was discarded, and 1 ml of 70% ethanol was added to the precipitate. After the microtubes were centrifuged at 10,000 rpm, 4°C for 4 minutes. The pellet was dissolved in 30 μL of the TE buffer. 

**Gene All kit method:** The total DNA was also extracted using the Gene All kit (Gene All Biotechnology Company, South Korea) as per the instructions of the kit.

 The quantity and purity of the extracted tDNA were determined using a UV-Vis spectrophotometer (Eppendorf AG, Hamburg 22331, Germany) at 230, 260, 280, and 320 nm. The absorbance ratios of 260/280 and 260/230 were calculated for the evaluation of DNA purity and the detection of protein and non-protein contamination, respectively [34,35]. The DNA samples were analyzed by electrophoresis using a 0.8% agarose gel [36]. Experiments were performed in triplicate. 

The quality of the tDNA extracted from the fresh and dried leaves of the studied herbs was examined by the PCR of the ITS fragments in the nrDNA and the *trn*L-F region in the chloroplast genome. Of the different *trn*L-F regions in the chloroplast, *trn*L _(UAA)_ (c) and *trn*F _(GAA)_(f) were analyzed [31]. Table 1 shows the sequences of the primers used and the size of the amplicons. In some cases, touchdown (TD) PCR was performed to increase the sensitivity and efficiency of the results [37]. 

**Table 1:** The sequence of universal primers used

	**Name**	**Sequence**	**Amplicon size (bp)**	**References**
**Nuclear region**	ITS5m (F)	5′-GAAGGAGAAGTCGTAACAAGG-3′	700	[38]
	ITSF (R)	5′-TCCTCCGCTTATTGATATGC-3′		[39]
**Chloroplast region**	trn L (UAA)(c) (F)	5′-CGAAATCGGTAGACGCTACG-3′	1100	[29]
	trn F (GAA)(f) (R)	5′-ATTTGAACTGGTGACACGAG-3′		

F, forward; R, reverse; of the different *trn*L-F regions in the chloroplast, *trn*L _(UAA)_ (c) and *trn*F _(GAA)_ (f) were examined.

Tables 2 and 3 show the main PCR and TDPCR programs used to amplify the nuclear ITS fragments, respectively. The PCR program for the chloroplast *trn*L-F region was the same as the main PCR program used for the nuclear region, except for the annealing temperature, which was 57°C for the chloroplast sequences. For amplification of the target sequence, 500 ng of DNA was used per 20 µl reaction volume containing 10 µl of 2X Ampliqon Master Mix (Ampliqon company, Denmark, Cat. No. 180301, 150 µM Tris-HCl pH 8.5, 40 µM (NH_4_)_2_SO_4_, 3.0 µM MgCl_2_, 0.4 µM dNTPs, 0.05 units µL-1 Amplicon Taq DNA polymerase, inert red dye, and a stabilizer) and 10 picomoles of each forward and reverse primer. Five μL of the PCR products were electrophoresed on a 1.5% agarose gel. The size of the PCR products on the agarose gel was determined using a DNA molecular weight marker.

**Table 2:** The main polymerase chain reaction program for amplifying the nrDNA ITS

**Steps**	Temprature (°C)*	**Time**	**Cycle(s)**
Initial denaturation	94	2 min and 30 sec	1
Denaturation	94	50 sec	30
Annealing	55, 56, or 58	30 sec	30
Extension	72	50 sec	30
Final extension	72	7 min	1

The same PCR program was used to amplify the chloroplast *trn*L-F regions. However, the annealing temperature for the chloroplast sequences was 57°C. *For the DNA extracted from fresh and dried leaves of the studied herbs, the following annealing temperatures were used: main CTAB (55°C), first and second modified CTAB (56°C), and modified Murray and Thompson methods and Gene All kit methods (58°C). (For more information on the DNA extraction methods, please refer to marials and methods section.)

**Table 3:** Touchdown polymerase chain reaction programs (TD 1 and 2 PCR) were used to amplify the nrDNA ITS

	**TD 1 PCR**				**TD 2 PCR**		
**Steps**	**Temperature (C°)**	**Time**	**Cycle(s)**		**Temperature (C°)**	**Time**	**Cycle(s)**
**Initial denaturation**	94	90 sec	1		94	90 sec	1
**First Denaturation**	94	50 sec	10		94	50 sec	10
**First Annealing**	56 - 58	30 sec	10		60 - 69	30 sec	10
**First Extension**	72	50 sec	10		72	50 sec	10
**Second Denaturation**	94	50 sec	32		94	50 sec	25
**Second Annealing**	57	30 sec	32		59	30 sec	25
**Second Extension**	72	50 sec	32		72	50 sec	25
**Final extension**	72	420 sec	1		72	420 sec	1

## RESULTS

There was no significant difference between the tDNAs extracted from both fresh and dried leaves of the studied herbs using each of the five extraction methods (Table 4). When the results of all extraction methods were compared (Table 4), no significant difference was observed between the studied extraction methods for the DNA extracted from fresh or dried leaves of the herbs in terms of the A260/A280 ratio. However, the difference between the DNA extracted from the fresh and dried leaves of *P. harmala* by the five extraction methods was significant in terms of the concentration of the tDNA and the A260/A230 ratio. For *T. ramosissima*, a significant difference between the extraction methods was found only in terms of the A260/A230 ratio for the dried leaves. In comparison, only the tDNA extracted from the dried leaves of *P*. *reptans* by the methods studied showed significant differences in terms of both the concentration of the tDNA and the A260/A230 ratio. For a more detailed investigation, the paired comparisons were made with the Mann-Whitney test which has been shown in Table 5.

**Table 4:** Statistical analysis of the concentration of the total extracted DNA (ng/µl) and the ratio of optical absorbance in the studied herbs

**The Kruskal-Wallis / The Mann-Whitney test**	***Peganum harmala*** ** L.**			***Tamarix ramosissima*** ** Ledeb**			***Potentilla reptans*** ** L.**		
for comparison of the DNA extracted from	**A260/A280**	**A260/A230**	**[DNA] (ng/µL)**	**A260/A280**	**A260/A230**	**[DNA] (ng/µL)**	**A260/A280**	**A260/A230**	**[DNA] (ng/µL)**
Fresh leaves by 5 extraction methods	0.079	**0.044**	**0.043**	0.191	0.244	0.180	0.204	0.055	0.051
Dried leaves by 5 extraction methods	0.096	**0.041**	**0.041**	0.104	**0.032**	0.324	0.068	**0.034**	**0.034**
Fresh and dried leaves using the CTAB method	1.000	0.100	0.100	0.200	1.000	1.000	0.333	0.333	0.333
Fresh and dried leaves using the first modified CTAB method	0.100	1.000	0.100	0.100	0.100	0.400	0.200	1.000	0.200
Fresh and dried leaves using the second modified CTAB method	0.800	0.200	1.000	0.200	0.200	0.400	0.100	0.100	0.700
Fresh and dried leaves using the modified Murray and Thompson method	0.100	1.000	0.700	1.000	0.700	0.700	0.100	0.100	0.700
Fresh and dried leaves using Gene All kit	0.667	1.000	0.333	1.000	1.000	0.667	0.667	0.667	0.667

The numbers in bold indicate that the difference is significant. The significance level was set at P<0.05.

**Table 5:** Statistical analysis of the concentration of the total extracted DNA ([DNA]; ng/µl) and the ratio of A260/A230 in groups with significant differences

	***Peganum harmala*** ** L.**				***Tamarix ramosissima*** ** Ledeb**	***Potentilla reptans*** ** L.**	
**Comparisons between methods **	**A260/A230 Fresh leaves**	**A260/A230 Dry leaves**	**[DNA]** (ng/µL) **Dry leaves**	**[DNA]** (ng/µL) **Fresh leaves**	**A260/A230 Dry leaves**	**A260/A230 Dry leaves**	**[DNA]** (ng/µL) **Dry leaves**
**CTAB and first modified CTAB **	**0.046**	**0.046**	**0.049**	0.127	**0.049**	0.139	0.248
**CTAB and second modified CTAB**	**0.049**	1	0.564	**0.046**	**0.083**	0.564	0.248
**CTAB and modified Murray and Thompson**	**0.049**	**0.049**	**0.046**	0.513	0.376	0.083	0.083
**CTAB and Gene All kit**	1	0.083	0. 564	0.083	0.083	0.121	0.121
**first modified CTAB and second modified CTAB**	**0.046**	0.076	0.083	0.825	0.248	0.127	0.127
**first modified CTAB and modified Murray and Thompson**	0.825	0.487	**0.046**	0.127	**0.049**	**0.049**	**0.049**
**first modified CTAB method and Gene All Kit**	0.197	0.236	0.083	0.083	0.083	0.083	0.083
**second modified CTAB and modified Murray and Thompson**	0.127	0.083	0.076	**0.046**	0.083	**0.049**	**0.049**
**second modified CTAB and Gene All Kit**	0.083	0.121	1	0.076	0.121	0.083	0.083
**modified Murray and Thompson and Gene All Kit**	0.248	0.083	0.076	0.083	0.083	0.083	0.083

The numbers in bold indicate that the difference is significant. The significance level was set at P<0.05.

In the next step, the results of the five extraction methods that showed a significant difference were compared to determine the best method for DNA extraction from fresh or dried leaves of each herb. As shown in Table 6, for the herb *P. harmala*, the highest and lowest concentrations of the tDNA extracted from the fresh leaves were obtained using the modified Murray and Thompson method and the Gene All kit, respectively. In comparison, the first and second modified CTAB methods gave the highest and lowest concentrations of the tDNA extracted from dried leaves, respectively. As for the A260/A230 ratio of the tDNA extracted from the fresh leaves, the second modified CTAB and the main CTAB methods resulted in the lowest and highest non-protein contaminants. However, for the dried leaves of *P*. *harmala*, the modified Murray and Thompson method and the main CTAB method ranked first and last, respectively. 

In *T. ramosissima*, the second modified CTAB method and the Gene All kit resulted in the highest and lowest A260/A230 ratio (Table 6). The main CTAB method gave the highest tDNA concentration. However, the Gene All kit found the lowest tDNA concentration and the highest non-protein contamination For the dried leaves of *P. reptans*, where the difference between the extraction methods was significant, the modified Murray and Thompson method and the Gene All kit resulted in the highest and lowest tDNA concentrations. The tDNA extracted from dried leaves of *P. reptans* using the Gene All kit also had the lowest A260/A230 ratio. However, the highest A260/A230 ratio was obtained with the first modified CTAB method (Table 6). 

**Table 6:** Ranking of the studied DNA extraction methods based on the concentration of the extracted total DNA ([DNA]) and optical absorbance ratios in the herbs *Peganum harmala*, *Tamarix ramosissima* and *Potentilla reptans*

		**Fresh leaves**	**Dried leaves**
***P. harmala***	**[DNA]** (ng/µL)	Modified Murray and Thompson method > Main CTAB > First modified CTAB > Second modified CTAB > Gene All kit	First modified CTAB > Modified Murray and Thompson method > Gene All kit > Main CTAB > Second modified CTAB
	**A260/A230**	Second modified CTAB > Modified Murray and Thompson method > First modified CTAB > Gene All kit > Maim CTAB	Modified Murray and Thompson method > First modified CTAB > Gene All kit > Main CTAB > Second modified CTAB
***T. ramosissima***	**A260/A230**	No significant difference was seen between the studied extraction methods	Second modified CTAB > First modified CTAB > Main CTAB > Modified Murray and Thompson method > Gene All kit
***P. reptans***	**[DNA]** (ng/µL)	No significant difference was seen between the studied extraction methods	Modified Murray and Thompson method > First modified CTAB > Main CTAB > Second modified CTAB > Gene All kit
	**A260/A230**	No significant difference was seen between the studied extraction methods	First modified CTAB > Second modified CTAB > Main CTAB > Modified Murray and Thompson method > Gene All kit

Table 7 shows the statistical comparison of the quantitative and qualitative parameters of the extracted tDNA between the studied herbs. The statistical comparison was performed based on the Kruskal-Wallis test, as explained in the data presented in Table 4.

The concentrations of the DNA extracted from the fresh and dried leaves of *P. harmala* by the five extraction methods were significantly different (p=0.043 and 0.041, respectively; Table 4). As shown in Table 7, the highest and lowest DNA concentrations were obtained from fresh leaves using the modified Murray and Thompson method (403.33±261.74) and the Gene All kit (51.50±3.53), respectively. The highest and lowest DNA concentrations for the dried leaves were extracted using the first modified CTAB (308.66±71.16) and the second modified CTAB (63.50±45.96), respectively.

The difference between the studied extraction methods was also significant with respect to the A260/A230 ratio between the extracted DNA from fresh and dried leaves of *P. harmala* (both p= 0.04; Table 4). The highest and lowest ratios of A260/A230 for the fresh leaves were obtained using the second modified CTAB method (0.72±0.04) and the modified Murray and Thompson method (0.54±0.02), respectively. For the dried leaves, the highest and lowest A260/A230 ratios were obtained using the modified Murray and Thompson (0.64±0.02) method and the second modified CTAB (0.45±0.12) method, respectively (Table 7).

**Table 7:** Statistical comparison of the quantitative and qualitative parameters of the extracted total DNA and PCR results of the nrDNA ITS and *trn*L-F (f and c) regions between the herbs *Peganum harmala*, *Potentilla reptans*, and *Tamarix ramosissima* Ledeb

	***P. harmala***		***T. ramosissima***		***P. reptans***		
	Fresh leaves	Dried leaves	Fresh leaves	Dried leaves	Fresh leaves	Dried leaves	
**The highest [DNA]** (ng/µL)	**Modified Murray and Thompson method** (403.33±261.74)*	**First modified CTAB** (308.66±71.16) *	Main CTAB method (236.00±112.6)	Main CTAB method (223.33±102.83)	Modified Murray and Thompson method (1131.3±541.47)	**Modified Murray and Thompson method** (1477.66±231.70) *	
**The lowest [DNA]** (ng/µL)	**Gene All kit** (51.50 ± 3.53)*	**Second modified CTAB** (63.50± 45.96)*	Gene All kit (46.00 ± 28.28)	Gene All kit (60.00±11.31)	Gene All kit (55.50±7.77)	**Gene All kit** (56.50 ± 7.77) *	
**The highest A260/A230**	Second modified CTAB method(0.72± 0.04) *	**Modified Murray and Thompson method** (0.64 ± 0.02)*	Second modified CTAB method (1.21 ± 0.300)	**Second modified CTAB** (1.77± 0.212) *	First modified CTAB method (1.45±0.742)	**First modified CTAB** method (1.66 ± 0.113) *	
**The lowest A260/A230**	**Main CTAB method** (0.54± 0.015) *	**Second modified CTAB** (0.45 ± 0.12) *	Gene All kit (0.46 ± 0.035)	**Gene All kit** (0.48± 0.070)*	Gene All kit (0.58±0.084)	**Gene All kit** (0.56 ± 0.070) *	
**A260/A280** **Between 1.8-2**	-	-	-	Second modified CTAB method (1.77± 0.212)	-	Main CTAB method (1.50± 0.02) First modified CTAB (1.66± 0.113) Second modified CTAB (1.51± 0.043)	
**The highest A260/A280**	Second modified CTAB method (1.35 ± 0.06)	Second modified CTAB method (2.06 ± 1.04)	Modified Murray and Thompson method (1.48 ± 0.129)	Second modified CTAB method (1.67 ± 0.042)	Main CTAB method (1.33±0.049)	Main CTAB method (1.51 ± 0.021)	
**The lowest A260/A280**	Modified Murray and Thompson method (1.19 ± 0.045)	Gene All kit (1.21 ± 0.084)	Gene All kit (1.28 ± 0.070)	Main CTAB method (1.25 ± 0.170)	Modified Murray and Thompson method	Gene All kit (1.11 ± 0.106)	
**A260/A280≥ 1.8**	-	Second modified CTAB method (2.06 ± 1.04)	-	-	-	-	
**A260/A280 between 1.5-1.8**	-	First modified CTAB method (1.70 ± 0.17)	-	Second modified CTAB method (1.67 ± 0.042)	-	Main CTAB method (1.51 ± 0.021) The first modified CTAB (1.50 ± 0.077)	

The asterisk indicates the elements where the extraction methods differed significantly based on the Kruskal-Wallis test.

Regarding the A260/A280 ratio, no significant difference was found between the extraction methods for fresh or dried leaves of *P. harmala* (p=0.079 and 0.096, respectively; Table 4). As can be seen in Table 7, the highest A260/A280 ratios for both fresh and dried leaves were obtained using the second modified CTAB method (1.35±0.06 and 2.06±1.04, respectively). However, the modified Murray and Thompson method and the Gene All kit gave the highest (1.19±0.045) and lowest (1.21±0.084) A260/A280 ratios for fresh and dried leaves, respectively. 

For *T. ramosissima*, no significant difference was found between the extraction methods with respect to the parameters studied, except for the A260/A230 ratio in the dried leaves (p= 0.032; Table 4). The highest amount of the DNA extracted from both fresh (236.00± 112.66) and dried leaves (223.33±102.83) was obtained by the main CTAB method. In comparison, the Gene All kit yielded the least amount of DNA from both fresh (46.00±28.28) and dried leaves (60.00 ±11.31).

The only parameter that changed significantly for *T. ramosissima* was the A260/A230 ratio in the dried leaves, yielding the highest (1.77±0.212) and lowest (0.48±0.070) ratios with the second modified CTAB method and the Gene All kit, respectively. For the fresh leaves, the highest (1.21±0.300) and lowest (0.46±0.035) ratios of A260/A230 were obtained with the second modified CTAB method and the Gene All kit, respectively.

The ratio of A260/A280 of the DNA extracted from *T. ramosissima* did not change significantly across the extraction methods (Table 4). The highest ratio in the fresh (1.48± 0.129) and dried (1.67±0.042) leaves of *T. ramosissima* was obtained using the modified Murray and Thompson method and the second modified CTAB method. However, the Gene All kit and the main CTAB method gave the lowest A260/A280 ratio in the fresh (1.28±0.070) and dried (1.25±0.170) leaves, respectively. 

For *P. reptans*, the five extraction methods differed significantly in the concentration and A260/A230 ratio of the DNA extracted from the dried leaves (both p=0.034), but not for the fresh leaves (Table 4). According to our results, the concentration of the DNA extracted from *P. reptans* leaves was higher compared to the other two herbs studied. As shown in Table 7, the highest DNA concentration was obtained in both the fresh (1131.33±541.47) and dried (1477.66±231.70) leaves using the modified Murray and Thompson method. The Gene All Kit, on the other hand, gave the lowest DNA concentration in both fresh (55.50±7.77) and dried (56.50±7.77) leaves. The Gene All kit also gave the lowest A260/A230 ratio in both the fresh (0.58±0.084) and the dried (0.56±0.070) leaves. However, the highest A260/A230 ratio was obtained by the first modified CTAB method in both fresh (1.45±0.742) and dried (1.66±0.113) leaves. 

Regarding the ratio of A260/A280 in the fresh or dried leaves of *P. reptans*, no significant change was observed across the extraction methods (p=0.204 and 0.068, respectively; Table 4). The highest ratio in the fresh (1.33±0.049) and dried (1.51±0.021) leaves was obtained by the main CTAB method. In comparison, the highest A260/A280 ratios in the fresh (1.33± 0.049) or dried (1.17±0.037) leaves were obtained by the main CTAB method and the modified Murray and Thompson method respectively.

**Figure 1:**
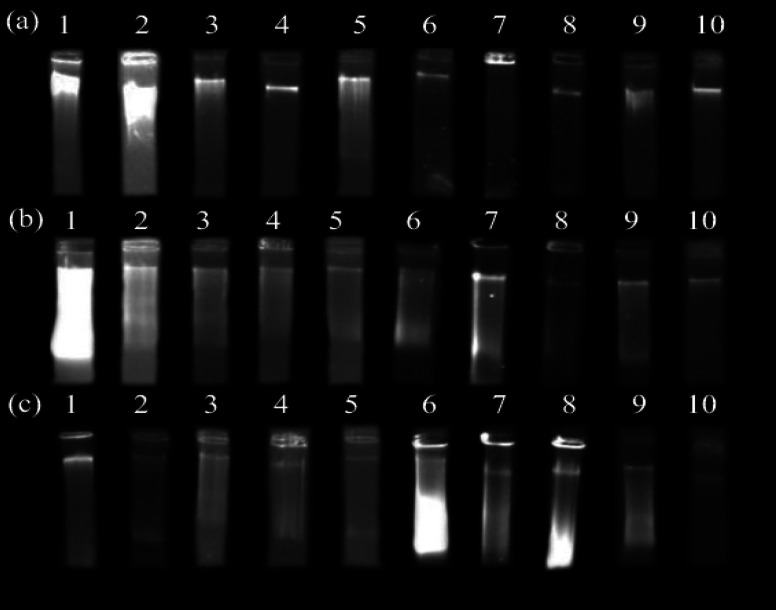
The quality assessment of the extracted total DNA from (a) *Peganum harmala* L., (b) *Tamarix. ramosissima* Ledeb., and (c) *Potentilla reptans* L. using the studied extraction methods. About 500 µg of DNA was analyzed by electrophoresis on a 0.8% agarose gel. 1) the main CTAB method - fresh leaves, 2) the main CTAB method - dried leaves, 3) the first CTAB method - fresh leaves, 4) the first CTAB method - dried leaves, 5) the second CTAB method - fresh leaves, 6) the second CTAB method - dried leaves, 7) the modified Murray and Thompson method - fresh leaves, 8) the modified Murray and Thompson method - dried leaves, 9) the Gene All kit- fresh leaves, and 10) the Gene All kit- dried leaves

Figure 1 shows the quality and integrity assessment results of the DNA samples extracted from the studied herbs by gel electrophoresis. As shown in Figure 1a, the tDNA extracted from the fresh and dried leaves of *P. harmala* by the first modified CTAB method had a good appearance compared to the other methods. For the fresh and dried leaves of *T. ramosissima* and *P. reptans*, the Gene All kit resulted in the best-quality DNA in appearance (Fig. 1b and c). 

**Figure 2:**
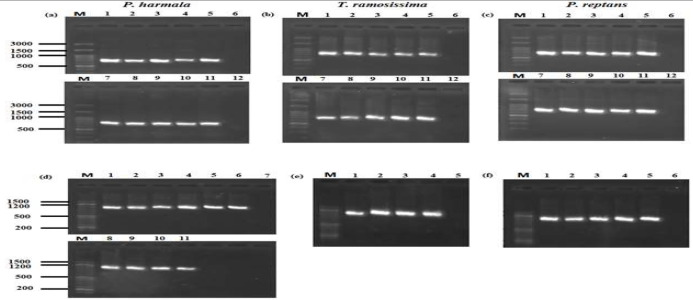
The results of the PCR of the ITS fragments in the nuclear ribosomal DNA of *Peganum harmala* L., *Tamarix ramosissima* Ledeb., and *Potentilla reptans* L. using the universal primers of ITS4 and ITS5m. M: DNA molecular ladder (DM2300), 1) the main CTAB method - fresh leaves, 2) the first CTAB method - fresh leaves, 3) the second CTAB method - fresh leaves, 4) the modified Murray and Thompson method - fresh leaves, 5) Gene All kit - fresh leaves, 6) negative control, 7) the main CTAB method - dried leaves, 8) the first CTAB method - dried leaves, 9) the second CTAB method - dried leaves, 10) the modified Murray and Thompson method - dried leaves, 11) the Gene All kit - dried leaves, and 12) negative control. (d, e, and f) The results of the PCR of the *trn*L-F region in the chloroplast genome of *P. harmala*, *T. ramosissima*, and *P. reptans*, respectively, using the universal primers of *trn*L(f) and *trn*L(c). M: DNA molecular ladder (DM2300); (d): 1) the Gene All kit - fresh leaves, 2) Gene All kit - dried leaves, 3) the second CTAB method - fresh leaves, 4) the second CTAB method - dried leaves, 5) the main CTAB method - fresh leaves, 6) the main CTAB method - dried leaves, 7) negative control, 8) the first CTAB method - fresh leaves, 9) the first CTAB method - dried leaves, 10) the modified Murray and Thompson method - fresh leaves, and 11) the modified Murray and Thompson method - dried leaves. (e): 1) Gene All - fresh leaves, 2) Gene All Kit - dried leaves, 3) the second CTAB method - fresh leaves, 4) the second CTAB method - dried leaves, 5) negative control. (f): 1) Gene All Kit - fresh leaves, 2) Gene All Kit - dried leaves, 3) the second CTAB method - dried leaves, 4) the first CTAB method - dried leaves, 5) the main CTAB method - dried leaves, 6) negative control

Table 8 shows the PCR programs used to amplify the ITS fragments in the DNA extracted from the leaves of the studied herbs. Figure 2 (a, b, and c) shows the results of the PCR of the ITS fragments of *P. harmala*, *T. ramosissima*, and *P. reptans*, respectively. The PCR products were amplified to the expected size (700 bp) from the tDNA extracted by all extraction methods studied. Figure 2 (d, e, and f) shows the results of the PCR of the *trn*L-F region in the chloroplast genome of *P. harmala*, *T. ramosissima*, and *P. reptans*, respectively. As expected, the PCR products were amplified at 700 bp with the tDNA extracted by all extraction methods examined. However, the PCR of the *trn*L-F region gave no results using the tDNA extracted by the main CTAB method, the first CTAB method, and the modified Murray and Thompson method from fresh or dried leaves of *T. ramosissima* when the main PCR programs (Table 6) or TD PCR (Table 7) were used.

**Table 8:** PCR programs used to amplify the nrDNA ITS in the DNA extracted from leaves of the herbs *Peganum harmala*, *Potentilla reptans*, and *Tamarix ramosissima* Ledeb

**DNA extraction method- plant sample**	**P. harmala**	**T. Ramosissima**	**P. reptans**
**Main CTAB method (Doyle and Doyle, 1987)** **fresh and dried leaves**	TD 2 PCR	Main PCR	Main PCR
**The first modified CTAB method - fresh leaves**	TD 2 PCR	TD 2 PCR	TD 1 PCR
**The first modified CTAB method - dried leaves**	Main PCR	Main PCR	Main PCR
**The second modified CTAB method - fresh leaves**	TD 2 PCR	TD 1 PCR	TD 1 PCR
**The second modified CTAB method - dried leaves**	Main PCR	Main PCR	Main PCR
**Modified Murray and Thompson method (Murray andThompson, 1980) fresh and dried leaves**	Main PCR	Main PCR	Main PCR
**Gene All kit - fresh leaves**	Main PCR	Main PCR	TD 2 PCR
**Gene All kit - dried leaves**	Main PCR	Main PCR	Main PCR

For more information on the main and touchdown polymerase chain reaction (TDPCR) programs, see Tables 2 and 3, respectively. For information on the extraction methods, see Section 2-2-DNA extraction.

## DISCUSSION

This is the first report on the extraction of the tDNA from medicinal plants suitable for molecular studies: *P. harmala*, *T. ramosissima*, and *P. reptans*. High-quality DNA extraction is essential for molecular studies and identification of the genetic source of herbal medicine and helps in ethnopharmacological and ethnobotanical studies [40]. DNA is often harder to extract from medicinal herbs because these plants usually contain more secondary metabolites, biochemicals other than DNA that interfere with the extraction process. In addition, different plants have different biochemical compositions [7, 8]. Therefore, the same methods will not work with all of them.

Young plant tissues are usually used for DNA extraction because they contain fewer polyphenols and polysaccharides compared with mature tissues. These substances interfere with DNA extraction and inhibit enzymatic digestion of DNA or PCR [9, 41, 42]. Drying can destroy cells and the cell wall, remove the cell membrane, and degrade the DNA. In addition, the sample of interest is sometimes a rare species, not easily found or is collected in remote areas. Therefore, it is kept in a dried or semi-dried state. Therefore, the usefulness of protocols for DNA extraction from dried samples cannot be overstated [41, 42]. 

In the present study, the tDNA was extracted from young fresh and dried leaves of the studied herbs using five methods: the main CTAB method [27] and its two modified protocols, the modified Murray and Thompson method [28, 29], and the Gene All kit. The first and second modified CTAB methods excluded BME and ammonium acetate, respectively, compared to the main method. The Murray and Thompson method was introduced in 1980 [28]. In the current study, a modified protocol of the Murray and Thompson method developed by Riahi et al. [29] was used, which does not require an ultracentrifuge.

For all herbs studied, there were no significant differences between the tDNAs extracted from fresh and dried leaves by each of the extraction methods examined in terms of concentration and optical absorbance ratios (Table 4). Considering the A260/A280 ratio, all extracted total DNAs from the fresh and dried leaves of all studied herbs had the same quality in terms of protein contaminants. However, none of the extraction methods studied resulted in an A206/A280 ratio greater than 1.8, except for the DNA extracted from the dried leaves of* P. harmala* (Table 7). It can be concluded that all the extracted DNAs had protein contaminants [35]. 

Significant differences were observed between the DNA extracted from the fresh or dried leaves of the studied herbs by the five extraction methods investigated in terms of DNA concentration and A260/A230 ratio (Table 4; results of the Kruskal-Wallis test). In the case of *P. harmala*, the tDNAs extracted from fresh and dried leaves differed significantly in terms of the A260/A230 ratio and concentration. The highest and lowest concentrations of the DNA extracted from fresh leaves were obtained using the modified Murray and Thompson method and the Gene All kit, respectively. In comparison, the DNA with the highest and lowest concentration was extracted from dried leaves using the first and second modified CTAB methods, respectively. The second modified CTAB method and the main CTAB method resulted in DNAs with the highest and lowest ratios of A260/230 extracted from the fresh leaves. However, the DNAs with the highest and lowest A260/230 ratios were extracted from the dried leaves using the modified Murray and Thompson method and the main CTAB, respectively. 

A significant difference between the studied extraction methods was only observed in the A260/A230 ratio of the tDNAs extracted from the dried leaves of *T. ramosissima*, such that the second modified CTAB and the Gene All kit resulted in the highest and lowest A260/230 ratios, respectively. 

For *P. reptans*, the concentration and A260/A230 ratio of the tDNAs extracted from only the dried leaves differed significantly across the methods studied. The modified Murray and Thompson method and the first modified CTAB method yielded the highest concentration and A260/230 ratio. However, the tDNA extracted using the Gene All kit had the lowest concentration and A260/230 ratio. (see Table 5).

However, only the *trn*L-F region in the chloroplast regions of the tDNA extracted from both the fresh and dried leaves of *P.harmala* by all methods examined was amplified by PCR (Fig. 2d). In comparison, the *trn*L-F region in the cpDNA was amplified only in the DNA extracted from the fresh and dried leaves of *T. ramosissima* using the second modified CTAB method and the Gene All kit (Fig. 2e). In *P. reptans*, the *trn*L-F region in the cpDNA was amplified only in tDNAs extracted both from the fresh and dried leaves using the Gene All kit and from the fresh leaves using the main and modified CTAB methods (Fig. 2f).

It appears that the removal of BME or ammonium acetate from the main CTAB as in the first and second modified CTAB methods, respectively, did not significantly affect the protein contaminants of the extracted DNAs, and the PCR results were acceptable. 

BME is a reducing agent that can remove tannins and polyphenols and denatures proteins by reducing disulfide bonds [43]. In many protocols, the addition of BME is an approach to improving the quality of the extracted DNA. Arruda et al. developed a modified protocol for DNA extraction from *Mimosa tenuiﬂora *(Willd.) Poir. In this method, which resulted in intact DNA successfully amplified in PCR, the concentrations of CTAB, NaCl, polyvinylpyrrolidone (PVP), and BME were increased, phenol was used to eliminate proteins, and the incubation time was decreased at lower temperatures [44].

Hammad and Qari [45] extracted DNA from some herbs related to *P. harmala*, such as *Zygophyllum coccineum* L., *Zygophyllum album* L.F., and *Zygophyllum aegyptium* A. Hosny using a modified CTAB method similar to the main CTAB method used in this study. They successfully used the extracted DNA for random amplified polymorphic DNA (RAPD).

The chloroplast genome (cpDNA) can provide a wealth of information on plant phylogeny, molecular ecology, population genetics, and evolution. The extraction of cpDNA and the nuclear genome usually reduces the quality of the cpDNA [46-48]. Therefore, the low quality or quantity of cpDNA in the extracted tDNA could be a possible reason for the observed PCR errors in the amplification of the chloroplast region in *T. ramosissima* and *P. reptans*. Not all research teams, though, have access to centrifuges or ultracentrifuges necessary for the isolation of intact chloroplasts and direct extraction of cpDNA from them.

In the present study, plant samples were homogenized without liquid nitrogen. Despite its great influence on the quality of DNA extraction, researchers performing DNA extraction often try to exclude liquid nitrogen from their protocols to save money, as tight research budgets do not allow such extravagances. Extreme care must also be taken when using this substance in a laboratory. It is highly toxic and poses a hazard to the user [49-51]. Ali et al., developed a method to extract DNA from Polianthes tuberosa using common laboratory equipment. The DNA was obtained in reasonable yield and was suitable for subsequent molecular studies [51].

This study is one of the few studies that have been statistically analyzed. However, it has several major shortcomings due to a lack of equipment and adequate research funding. The most important deficiency was the lack of detailed phytochemical analyses. We did not examine phytochemicals in any of the fresh or dried leaves. However, in other similar studies on these herbs, researchers have found useful information on *P*. *harmala* [11, 52], *T*. *ramosissima* [19, 21], and *P*.* reptans* [26, 53]. It is also worth noting that the nature of the remaining contaminants in the extracted DNAs was not studied. Protein and non-protein contaminants were evaluated only by calculating the ratios of A260/A280 and A260/A230, respectively. Accordingly, the exact reason for the observed PCR failures in some DNA samples of *T*. *ramosissima* and *P*. *reptans* was not determined. The second shortcoming is that only the extraction protocols were performed in three replicates. Therefore, it was impossible to analyze the results' normality and apply parametric methods. Despite all these shortcomings, this study is important for two reasons: 1) we statistically compared the results from five different extraction methods, and 2) the results apply to laboratories that have the same limitations as we do.

### Acknowledgements:

The authors would like to thank Damghan University for the financial support of this study.

### Conflict of Interest:

The authors declare that they have no known competing financial interests or personal relationships that could have appeared to influence the work reported in this paper.
